# Antibiotic prophylaxis for dental procedures: do dental students know enough?

**DOI:** 10.1080/07853890.2021.1897460

**Published:** 2021-09-28

**Authors:** Sara Manso, José João Mendes, P. Patrícia Cavaco Silva

**Affiliations:** ^a^Instituto Universitário Egas Moniz (IUEM), Egas Moniz Cooperativa de Ensino Superior, Caparica, Portugal; ^b^Centro de Investigação Interdisciplinar Egas Moniz (CiiEM), Egas Moniz Cooperativa de Ensino Superior, Caparica, Portugal

## Abstract

**Introduction:**

According to the American Heart Association (AHA) the cardiac conditions with the highest risk of infective endocarditis (IE) for which antibiotic prophylaxis (AP) is reasonable are: prosthetic cardiac valves, previous IE, congenital heart disease and cardiac transplantation recipients [1,2]. The aim of this study was to analyse the knowledge of AP in Dental Medicine students.

**Materials and methods:**

A prospective and analytical study was conducted using a questionnaire designed to describe the knowledge of AP and covering issues such as: AP guidelines, dental procedures and medical conditions needing AP, and recommended antibiotics. A 0–10 score was attributed to the questionnaire and the correct answers were based on AHA guidelines. The questionnaire was applied to students of the two last curricular years (4^th^ and 5^th^ year) of a Master in Dental Medicin and to newly graduated trainees at a University Dental Clinic – both in the Greater Lisbon area. The questionnaire was authorised by the students through a declaration of informed consent. This study was authorised by the Clinical Director of CDEM and approved by Egas Moniz Ethics Committee.

**Results:**

A total of 275 questionnaires were obtained with an answer rate of: 93.8%/4^th^ year (*n* = 135), 84.5%/5^th^ year (*n* = 120) year and 57.1%/trainees (*n* = 20). The median score of the questionnaire was 6.0, 6.4 and 6.8 for 4^th^ and 5^th^ year students and trainees, respectively. The results were more satisfactory regarding: guideline existence knowledge, high-risk conditions recommended for AP and antibiotic selection in non beta lactam allergic patients. Unsatisfactory answers were related to: dental procedures in need of AP, heart conditions associated with IE, and antibiotic selection in beta lactam allergic patients. Noteworthy, antibiotic selection and dental procedure knowledge was significantly better in postgraduates.

**Discussion and conclusions:**

The knowledge of the undergraduates and newly graduates concerning AP for dental procedures was not totally satisfactory, however there was a positive evolution according to the academic degree. As pharmacology subjects are taught in the early years of the course, antibiotic therapeutics should be reinforced later during the study cycle, and students should be made aware of the importance of a rational and adequate use of antibiotics in dental practice. There is a need to improve the knowledge and communication over this topic not only among undergraduates but also regarding postgraduates, in order to encourage clearer and more homogeneous antibiotic prescription patterns.
